# Understanding the burden of bacterial sexually transmitted infections and *Trichomonas vaginalis* among black Caribbeans in the United Kingdom: Findings from a systematic review

**DOI:** 10.1371/journal.pone.0208315

**Published:** 2018-12-07

**Authors:** Sonali Wayal, Catherine R. H. Aicken, Catherine Griffiths, Paula B. Blomquist, Gwenda Hughes, Catherine H. Mercer

**Affiliations:** 1 Institute for Global Health, University College London, London, United Kingdom; 2 HIV & STI Department, Centre for Infectious Disease Surveillance and Control (CIDSC), Public Health England, London, United Kingdom; 3 The National Institute for Health Research Health Protection Research Unit (NIHR HPRU) in Blood Borne and Sexually Transmitted Infections at UCL in partnership with Public Health England (PHE) and in collaboration with the London School of Hygiene & Tropical Medicine, London, United Kingdom; Monash University, AUSTRALIA

## Abstract

**Background:**

In the UK, people of black Caribbean (BC) ethnicity continue to be disproportionately affected by bacterial sexually transmitted infections (STIs) and *Trichomonas vaginalis* (TV). We systematically reviewed evidence on the association between bacterial STIs/TV and ethnicity (BC compared to white/white British (WB)) accounting for other risk factors; and differences between these two ethnic groups in the prevalence of risk factors associated with these STIs, sexual healthcare seeking behaviours, and contextual factors influencing STI risk.

**Methods:**

Studies presenting relevant evidence for participants aged ≥14 years and living in the UK were eligible for inclusion. A pre-defined search strategy informed by the inclusion criteria was developed. Eleven electronic databases were searched from the start date to September-October 2016. Two researchers independently screened articles, extracted data using a standardised proforma and resolved discrepancies in discussion with a third researcher. Descriptive summaries of evidence are presented. Meta-analyses were not conducted due to variation in study designs. Preferred Reporting Items for Systematic Reviews and Meta-Analysis (PRISMA) guidelines were followed.

**Results:**

Of 3815 abstracts identified, 15 articles reporting quantitative data were eligible and included in the review. No qualitative studies examining contextual drivers of STI risk among people of BC ethnicity were identified. Compared to the white/WB ethnic group, the greater STI/TV risk among BCs was partially explained by variations in socio-demographic factors, sexual behaviours, and recreational drug use. The prevalence of reporting early sexual debut (<16 years), concurrency, and multiple partners was higher among BC men compared to white/WB men; however, no such differences were observed for women. People of BC ethnicity were more likely to access sexual health services than those of white/WB ethnicity.

**Conclusions:**

Further research is needed to explore other drivers of the sustained higher STI/TV prevalence among people of BC ethnicity. Developing holistic, tailored interventions that address STI risk and target people of BC ethnicity, especially men, could enhance STI prevention.

## Introduction

In the United Kingdom (UK), sexually transmitted infections (STIs) continue to be a public health concern [1, 2]. Studies since the late-1980s have shown disproportionately high rates of bacterial STI diagnoses among black compared to white ethnic groups [3, 4]. Since 2000, clinic-based studies [5, 6], national probability surveys [7, 8], and surveillance data [9, 10] have distinguished between ‘black Caribbean’ (BC), ‘black African’ (BA) and ‘black other’ (BO) ethnicities, and have consistently shown that people of BC ethnicity in particular experience the highest rates of infection with *Trichomonas vaginalis* (TV) and bacterial STIs. Rates of gonorrhoea are 8–12 times higher among people of BC ethnicity compared to people of white ethnicity [9, 11]. Age and sex related variations exist in rates of gonorrhoea and chlamydia diagnoses across and within ethnic groups, including among people of BC ethnicity [11]. The risks of co-infection and reinfection with bacterial STIs are also higher among BC compared to other ethnic groups [12–14].

Understanding the social patterning and determinants of poor sexual health is key to promoting equitable health and informing evidence-based public health policy and practice [8]. Given the high burden of bacterial STIs and TV among people of BC ethnicity in the UK, we undertook a systematic literature review to examine if ethnic variations in factors known to be associated with STIs/TV (for example: age) explain ethnic variations in STIs/TV at a population level. Specifically, we examined the evidence on the association between bacterial STI/TV and ethnicity (BC compared to white/white British (WB)) because the latter is the predominant ethnic group in the UK corresponding to approximately 90% of the population [15]. We also examined variations between these two ethnic groups in the prevalence of behavioural risk factors associated with STIs, sexual healthcare seeking behaviour, and contextual factors influencing STI risk.

## Methods

This research was conducted through the National Institute of Health Research Health Protection Research Unit (NIHR HPRU) in Blood Borne and Sexually Transmitted Infections at UCL in partnership with Public Health England and in collaboration with the London School of Hygiene & Tropical Medicine.

### Inclusion criteria

Studies examining the association between ethnicity (BC compared to white/WB) and bacterial STIs/TV were eligible for inclusion. We also included studies if the outcome variable considered bacterial and viral STIs together but predominantly comprised of bacterial STIs. Studies conducted only among people living in the UK were included because our focus was to understand the factors driving the sustained disproportionate burden of these infections among BC people in the UK. Studies examining variations in the prevalence of risk behaviours associated with these infections and in sexual healthcare seeking behaviours between these two ethnic groups, and studies exploring contextual drivers of STI risk among BC people were also eligible for inclusion. Studies conducted among persons aged ≥14 were eligible for inclusion because the rates of bacterial STI diagnoses increase substantially from the age of 14 [2].

### Exclusion criteria

Studies that did not differentiate between different ‘black’ ethnic groups were excluded due to variations in STI prevalence among different black communities, and because ‘black’ is a heterogeneous category, including for example, variations in history of migration, risk behaviours, and background STI prevalence in home countries among migrant populations [7]. Additionally, we excluded studies that met the inclusion criteria but provided scant data [16, 17], determined via discussion between two researchers. We also excluded studies not written in English.

### Search strategy

The following electronic databases were searched from the start up to September-October, 2016 (S1 Table) for empirical studies using a pre-defined search strategy informed by the review inclusion/exclusion criteria (S1 File): Medline, Embase, Cinahl, Psychinfo, Scopus, Web of Science, British Humanities Index, Applied Social Science Index and Abstracts, International Bibliography of the Social Sciences, Sociological Abstracts, and the Cochrane database of systematic reviews. Search terms were adapted to meet the requirements of different databases and search results imported into Endnote software. We also contacted two researchers in the field for unpublished papers/reports.

### Screening and data extraction

Following merging and deduplication of search results, two researchers independently screened 5% of all titles and abstracts to develop consensus for inclusion of studies, using pre-specified screening questions (S2 Table) which were informed by the inclusion/exclusion criteria. Subsequently a researcher screened the remaining titles and abstracts. Reference lists of studies included in the review were also screened. Once the eligible papers were identified, data were extracted and quality appraisal was conducted independently by two researchers for five of the included studies to pilot the data extraction pro-forma (S2 File) and quality appraisal pro-forma (S3 Table) and checked for concordance. The quality appraisal pro-forma was adapted from the NICE guidance for ‘quality appraisal checklist for quantitative studies reporting correlations and associations’ [18]. This checklist seeks to assess the key population criteria for determining the study’s external validity, i.e. the extent to which the findings of the study are generalizable to the study’s source population. It also seeks to assess the internal validity of the study using various criteria, i.e., that the study has been carried out carefully and the identified associations are valid. Disagreements regarding study inclusion and discrepancies in data extraction and quality appraisal were resolved through discussion with a third researcher. Subsequently data extraction and quality appraisal of all the remaining papers was conducted by a researcher and was checked by another researcher.

### Data synthesis

Quantitative studies included in the review varied in study design, methods, definitionsand measurement of outcomes and explanatory variables. Thus a meta-analysis was not conducted to avoid the risk of deriving misleading conclusions [19], and instead we conducted a narrative synthesis of evidence for each of our research questions. Studies were analysed according to the type of STIs, and study design. Descriptive summaries are presented on the reported evidence relating to (i) the association between ethnicity (BC vs white/WB) and STIs/TV after adjusting for other factors, and (ii) variations in the prevalence of risk behaviours associated with STIs/TV, and (iii) variations in sexual healthcare seeking behaviours between these two ethnic groups.

We followed the Preferred Reporting Items for Systematic Reviews and Meta-Analysis (PRISMA) guidelines (S4 Table) [20].

## Results

**Fig 1:**
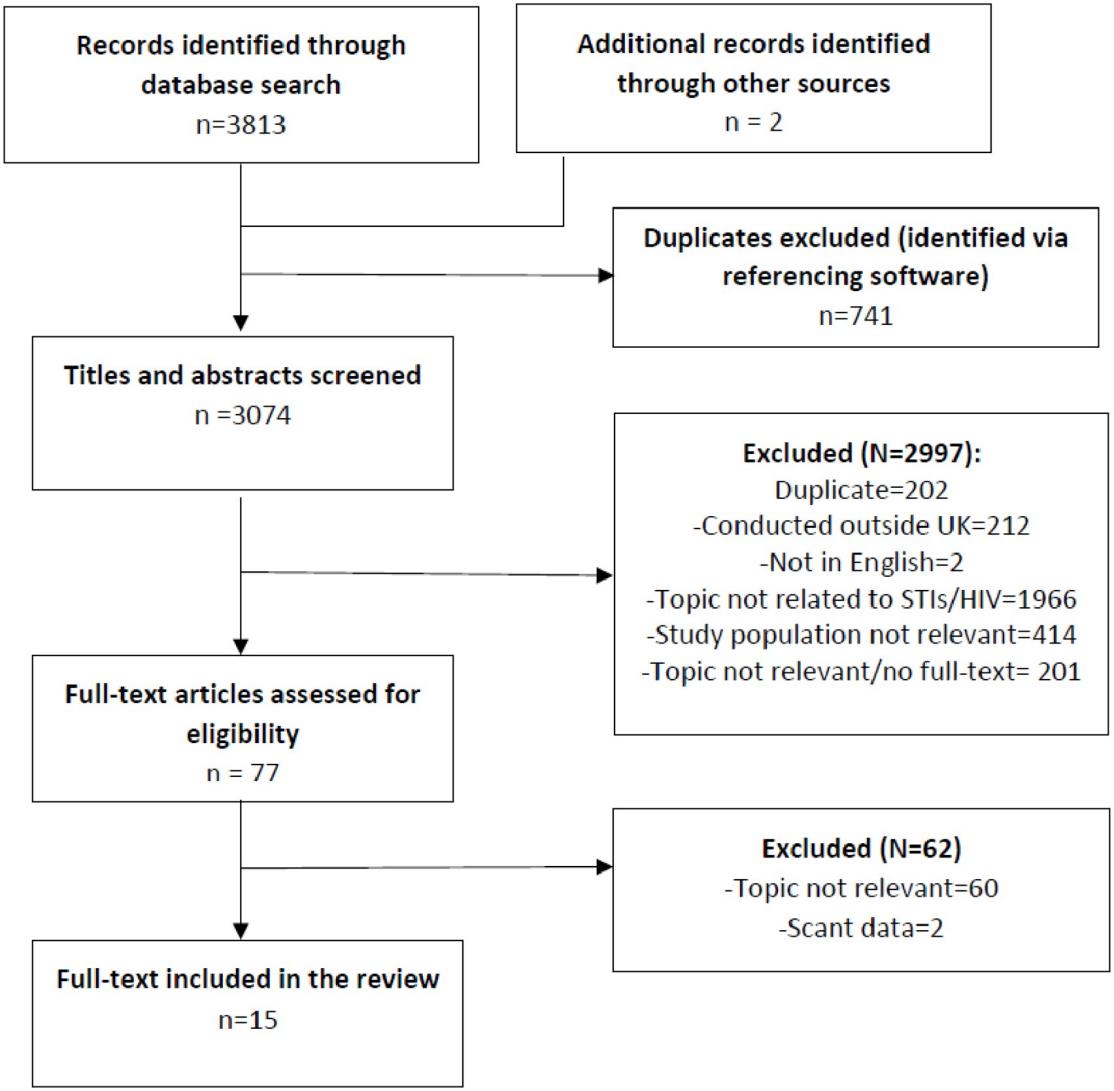
Flow diagram of records screening for identifying eligible studies.

We identified 3815 records (Fig 1). Fifteen quantitative studies were eligible and included in the review. Of these 13 studies were identified during electronic searches, one through screening of the reference list of an included paper, and one was a paper published by our research team which was under review when we conducted electronic database searches and was subsequently published in 2017 (Table 1). No qualitative studies that examined contextual drivers of STI risk only among people of BC ethnicity were identified.

**Table 1:** Description of studies included in the review.

Authors	Study focus	Aims	Study design	Population, setting & year (number of individuals/episodes/attendances/tests)	Methods	Ethnic groups	Quality grading for internal validity	Quality grading for external validity
Dragovic, 2002 [12]	Gonorrhoea and Chlamydia	To examine co-infection with chlamydia in patients diagnosed with gonorrhoea and examine risk factors for co-infection.	Case note review	All patients diagnosed with gonorrhoea in 3 sexual health clinics in West London, April-September 1998 (total n = 153, BC n = 32)	Demographic and laboratory results data (from culture/ELISA test) were extracted using a proforma, for all gonorrhoea cases.	Black Caribbean, Black African, White, Other/ Unknown.	+	+
Fenton, 2005 [7]	Any STI	To investigate ethnic variations in high risk sex behaviour and sexual health outcomes in Britain and their association with key demographic and behavioural factors.	Cross-sectional	Men and women aged 16–44 in Britain, 1999–2001 (total n = 11,161; BC n = 225)	Multistage probability sampling using postcode address file, with oversampling of areas of high density of four ethnic minority groups (Black Caribbean, Indian, Pakistani, Black African). Survey conducted using computer-assisted personal interviews and self-interviews.	Black Caribbean, Black African, Indian, Pakistani, White	++	++
Furegato, 2016 [9]	Gonorrhoea, Syphilis	To investigate the association between ethnicity, STI diagnosis rate and SED (socioeconomic deprivation) in England.	Cross-sectional	All sexual health clinic attendances in England, 2013 (total attendances n = 2,539,572; BC n not reported)	Data on all STI diagnosis made in sexual health clinics in England in 2013 was extracted from routine STI surveillance system (GUMCAD) along with clinical and socio-demographic data on each patient attendance.	Black Caribbean, Black African, Black other, Asian, Indian, Pakistani, Bangladeshi, Chinese, Asian other, White British, White Irish, White other, mixed ethnicity, other ethnicity	++	++
Gerressu, 2012 [26]	Sexual healthcare seeking behaviours	To explore patterns of care-seeking behaviour for STIs separately for Black Caribbeans and Black Africans.	Cross-sectional	All new patients attending 7 sexual health clinics in England, October 2004-March 2005 (total n = 2824, BC n = 345)	Sexual health clinics were selected to represent different demographic, geographic, service configuration characteristics that can influence sexual health need and service use. Questionnaire data were linked to clinics’ routinely-collected data on STI diagnoses associated with the same clinic attendance.	Black Caribbean, Black African, White	+	+
Hughes, 2001 [13]	Acute STIs*	To investigate the demographic and behaviour characteristics of sexually transmitted disease clinic patients most likely to re-attend with an STI.	Retrospective cohort	Patients diagnosed with an acute STI who re-attended within 1 year, at 3 sexual health clinics in England, 1994–1998 (total n = 17,446, BC n = 2,924)	Demographic and diagnosis data were collected from clinic databases. Behavioural data were recorded on proformas completed by the attending doctor.	White, Black Caribbean, Black African, Asian, Other/mixed	+	+
Hughes, Catchpole 2000 [6]	Gonorrhoea, Chlamydia	To compare the risk factors for four common sexually transmitted infections (STIs) in attenders at three large urban GUM clinics in England.	Cross-sectional	First attendances of patients attending 3 sexual health clinics in England participating in sentinel surveillance, 1st April 1994-30th September 1997 (total attendances n = 18,238, BC n not reported)	Sentinel surveillance data on STI diagnoses, demographic characteristics, and risk behaviours were collected for each patient attendance in the study period.	White, Black Caribbean, Black African, Asian, Other/Mixed (includes Black Other)	+	+
Hughes, 2013 [14]	Gonorrhoea	To estimate risk and important socio-demographic and behavioural determinants of gonorrhoea reinfection.	Cross-sectional	Sexual health clinic patients diagnosed with gonorrhoea within 42 days of previous diagnosis, Sheffield, 2004–2008 (total n = 1,650, BC n = 141)	Data on gonorrhoea diagnosis, other clinical data, demographic and behavioural data were extracted from patients’ clinical records.	White, Black Caribbean, Black African, Asian, Other/mixed	+	+
Jayakody, 2011 [25]	Behavioural risk factors	To determine how ethnic background influences early sexual activity among young adults.	Longitudinal cross-sectional surveys	Male and female students attending secondary schools in East London (totals: Phase 1: 11–14 year olds; n = 2,790; BC n not reported and Phase 2: 13–16 year olds, n = 2,675; BC n = 156)	30/43 secondary schools were randomly selected and balanced to ensure representation of single-sex & mixed-sex schools. Questionnaire survey administered in school setting. Sexual behaviour data were collected at Phase 2 (age 13–16).	Black Caribbean, Black African, Black British, White British, White Other, Bangladeshi, Indian, Pakistani, Mixed Ethnicity, Other	+	+
LaMontagne 2004 [21]	Chlamydia	To describe the implementation of the English National Chlamydia Screening Programme (NCSP) first year, positivity rates, and risk factors for genital chlamydial infection.	Cross- sectional	Chlamydia tests among sexually active young people aged <25 years, screened through England’s NCSP, April 2003-June 2004 (total n = 16,413 chlamydia tests; BC n = 413)	Data on each screening test (including test results and socio-demographic variables) was collated from routinely-collected data.	White, Black Caribbean, Black African, Black British-Other, Asian subcontinent, Chinese—other Asian, Other ethnic group, mixed/unknown	+	+
Low, 2001 [11]	Chlamydia & Gonorrhoea	To examine differences in population based rates of gonorrhoea and chlamydia between Black ethnic groups in Lambeth, Southwark, and Lewisham Health Authority.	Cross-sectional	Episodes of gonorrhoea (among men and women) or chlamydia (among women) recorded among attendees at 11 sexual health clinics in South London, 1 January 1994 to 31 December 1995 (totals: n = 1996 gonorrhoea episodes; n = 1376 chlamydia episodes; among BC: n = 966 gonorrhoea episodes; n = 608 chlamydia episodes)	Episodes of infection were identified from sexual health clinic records, using standardised codes that are used for surveillance reports and residence in the study area was determined from the postcodes for Lambeth, Southwark, and Lewisham Health Authority areas. Data on chlamydia was collected only for women because testing in men was not routinely done in all clinics.	Black Caribbean, Black African, Black other, Asian/other, White	+	+
Mitchell, 2014 [10]	Trichomonas vaginalis	To investigate the distribution and risk factors of *Trichomonas vaginalis* infection in England.	Cross-sectional	All sexual health clinic attendances between 2009–2011, by individuals resident in England (total n = 3,221,854 attendances; BC n not reported)	Clinical and socio-demographic data on first episode of diagnosis with *Trichomonas vaginalis* (for cases) or first sexual health clinic attendance (for patients without TV diagnosis) was extracted from routine STI surveillance system (GUMCAD).	White, Black Caribbean, Black African, Asian, Black Other, Other	++	++
Radcliffe, 2001 [22]	Chlamydia	To investigate demographic and behavioural risk factors associated with chlamydial infection among attendees at a large sexual health clinic in Birmingham, UK.	Case-control	All men and women attending a sexual health clinic in Birmingham between 14 June 1997–13 June 1998 presenting as a new clinical episode and willing to screen for both gonorrhoea and chlamydia. Cases were those who were diagnosed with chlamydia but not gonorrhoea. Controls were randomly selected from patients who were free of both infections (total n = 986 cases; 1212 controls; BC n = 397 cases; 265 controls).	Structured patient questionnaire.	Black Caribbean, White, other	+	+
Sheringham, 2011 [23]	Chlamydia	To examine variations in NCSP delivery and risk of screening positive for chlamydia in men and women by socioeconomic circumstances and age.	Cross-sectional	Chlamydia tests among sexually-active young people aged 13–24 years, screened through England’s NCSP, 1st January–31st December 2008 (n = 331,294; BC n not reported)	Data on each screening test (and socio-demographic variables) offered was collected, excluding sexual health clinics. These records were linked to Index of Multiple Deprivation 2007 using the National Statistics Postcode Directory.	White, Black Caribbean, Black African, Black background unspecified, Indian, Pakistani, Bangladeshi, Asian background unspecified, Chinese/other, Mixed	+	+
Simms, 2009 [24]	Chlamydia	To examine variation in positivity within the English NCSP during 2007/08.	Cross-sectional	Chlamydia tests among sexually active young people aged <25 years, screened through England’s NCSP, 2007–2008 (total n = 334,902 chlamydia tests; BC n = 8823)	Data on each screening test (including test results and socio-demographic variables) between 2007–2008 was collated, including screens offered in a range of settings but not sexual health clinics.	White, Black Caribbean, Black African, Black background unspecified, Indian, Pakistani, Bangladeshi, Asian background unspecified, Chinese/other, Mixed, Unknown	+	+
Wayal, 2017 [8]	Any STI	To investigate ethnic differences in hypothesised explanatory factors such as socioeconomic factors, substance use, depression, and sexual behaviours, and whether they explain ethnic variations in sexual health markers.	Cross-sectional	Men and women aged 16–74 in Britain, 2010–2012 (total n = 14,563; BC n = 178)	Multistage probability sampling using postcode address file. Survey conducted using computer-assisted personal interviews and self-interviews.	Black Caribbean, Black African, Indian, Pakistani, White British, White other, Mixed ethnicity	++	++

-Abbreviations: NCSP, National Chlamydia Screening Programme; NA, not applicable;

*Acute STI diagnosis was defined as: syphilis (primary and secondary infections), gonorrhoea, genital chlamydia, non-specific urethritis, trichomoniasis, chancroid/LGV/Donovanosis, genital warts (1st episode), genital herpes (1st episode), molluscum contagiosum, or scabies/pediculosis;

'++' Indicates that for the stated external or internal validity checklist question, the study has been designed or conducted in such a way as to minimise the risk of bias; '+' Indicates that either the answer to the checklist question is not clear from the way the study is reported, or that the study has not addressed all potential sources of bias for that particular aspect of study design.

### Characteristics of included studies and participants

Of the 15 studies included in the review (Table 1), ten examined risk factors either for single or multiple STIs accounting for ethnicity [6–11, 21–24]. Of these, most studies examined the risk factors for chlamydia or gonorrhoea, but a few examined risk factors for TV [10], syphilis [9], or ‘any bacterial STIs’ [7, 8]. Furthermore, one study examined factors associated with the risk of gonorrhoea and chlamydia co-infection [12]. Another study examined the risk of re-infection with gonorrhoea [14], and a study examined the risk of acute STIs among patients re-attending a sexual health clinic within one year [13]. Differences between BC and white/WB ethnic groups in the prevalence of behavioural risk factors associated with bacterial STIs/TV were examined by three studies [7, 8, 25], and in sexual healthcare seeking behaviours by three studies [7, 25, 26].

Four of the 15 studies included only young people aged 14–24 years [21, 23–25]. All three studies examining ethnic variations in behavioural risk factors and sexual healthcare seeking behaviours provided data on both sexes separately [7, 8, 25].

#### Study design

Two studies used data from national probability surveys in Britain (England, Scotland and Wales) [7, 8], and three used data from sexual health clinics in England only, comprising a cross-sectional survey [26], a case-control study [22], a retrospective case note review [12]. Six studies used routine or sentinel surveillance data on clinic attendees in England [6, 9–11, 13, 14]. Three studies used data from England’s National Chlamydia Screening Programme (NCSP) which targets sexually-active young people aged 14–24 years [21, 23, 24]. One study reported data from a cross-sectional survey in secondary schools in London among students aged 11–16 years [25].

### Quality assessment of included studies

As shown in S4 Table, of the 15 studies included in the review, the majority had reported data that enabled assessment of internal and external validity of the study results. As shown in Table 1 and S3 Table, the scores for internal and external validity of study results of four studies (two national probability surveys and two studies using surveillance data from all sexual health clinics in England) [7–10] were higher than for the other studies. For the other eleven studies, either the generalisability was limited (for example, due to sample selection or recruitment bias [21, 23, 24], or their internal validity was limited (for example, due to low participant response rate [22].

### Associations between ethnicity and STIs adjusting for other factors

Studies examined the association between ethnicity (BC compared to white/WB) and different types of bacterial STIs/TV diagnoses adjusting for a diverse range of factors (Table 2). Studies that used surveillance data showed that the association between ethnicity and diagnoses of gonorrhoea [6, 9, 11], chlamydia [6, 11], syphilis [9], and TV [10], was only partially explained by differences between BC and white/WB ethnic groups in their sociodemographic characteristics, including age, area-level deprivation, sexual orientation, and in previous STI diagnosis. Similarly, among participants screened for chlamydia for the English NCSP, the risk of chlamydia positivity among BC compared to white/WB young people continued to be higher despite accounting for socio-demographic factors and for screening venue [21, 23, 24]. This finding was also observed in a case-control study [22] and in studies that used national probability survey data which in addition to sociodemographic factors had adjusted for individual-level economic status and sexual behaviours [7, 8], and recreational drug use [8]. Taken together, these studies indicate that variations between BC and white/WB ethnic groups in a number of socio-demographic, economic and behavioural factors do not fully explain differences in STI prevalence between these ethnic groups.

**Table 2:** Association between bacterial STIs/*Trichomonas vaginalis* and ethnicity after adjusting for other factors.

Authors	Type of infection	Unadjusted OR/HR/RR/IRR for STIs (black Caribbeans vs. white/white British), 95% CI	Adjusted OR/HR/RR/IRR for STIs (black Caribbeans vs. white/white British), 95% CI	Other factors adjusted for
***Association between ethnicity and bacterial STIs/TV***				
Fenton, 2005 [7]	Any STI diagnosis in the last 5 years	ORs: Women: 2.41 (1.35–4.28) Men: 2.74 (1.22–6.15)	AORs: Women: 2.71 (1·41–5.21) Men: 2.66 (1.05–6.71)	Age*, residence in London (vs. rest of Britain), marital status, individual-level economic status, number of sexual partners in the last 5 years*, homosexual partner in the past 5 years^#^, new sex partners from outside the UK in the past 5 years^¶^, last sex was unprotected*, paid for sex in the past 5 years (data available from men only)^#^
Furegato, 2016 [9]	Gonorrhoea and Syphilis	IRRs: Men and women: Gonorrhoea: 8.18 (7.77–8.61) Syphilis 5.83 (4.77–7.13)	IRRs: Men and women: Gonorrhoea: 1.91 (1.82–2·02) Syphilis: 1.38 (1.13–1.70)	Age, area level deprivation (IMD)*, sexual orientation*
Hughes, Catchpole 2000 [6]	Gonorrhoea & Chlamydia	Not reported	AORs: Men: gonorrhoea: 4.32 (3.19–5.85) chlamydia: 2.16 (1.66–2.80) Women: gonorrhoea: 3.13 (2.06–4.76) chlamydia: 2.08 (1.62–2.68)	Age*, sexual orientation*^†^, number of partners in past 12 months*, previous STI, ever injected drugs, clinic (i.e. which of the three participating clinics the patient attended)*
LaMontagne, 2004 [21]	Chlamydia	OR: Screening tests among young women: 2.11 (1.61–2.78) Screening tests among young men: 3.32 (1.72–6.41)	AORs: Screening tests among young women: 2.04 (1.50–2.76) Screening tests among young men: 2.76 (1.29–5.93)	Age*, new sex partner (past 3 months)¶, >1 sex partner (past year)¶, specimen type (urine/cervical swab/vulvo-vaginal swab—relevant for women only), type of laboratory test*
Low, 2001 [11]	Gonorrhoea & Chlamydia	Not reported	Rate ratios: Women: gonorrhoea: 13.2 (10.7–16.2) chlamydia: 8.1 (7.1–9.3) Men: gonorrhoea: 11.6 (10.1–13.4)	Age*, area level deprivation (Townsend score)*
Mitchell, 2014 [10]	*Trichomonas vaginalis*	OR: Women: 8.56 (8.16–8.99) Men: 14.0 (11.9–16.5)	AORs: Women: 4.23 (3.98–4.50) Men: 8.00 (6.48–9.87)	Age*, region of birth*, area-level deprivation (IMD)*, Strategic Health Authority*, co-infection with acute warts*, co-infection with acute gonorrhoea¶ *In addition*, *in the model for women only*: co-infection with acute chlamydia¶, co-infection with acute herpes¶, bacterial vaginosis¶, candidiasis¶
Radcliffe, 2001 [22]	Chlamydia	Not reported	ORs: Men and women: 2.0 (1.5–2.7) Women: 1.9 (1.2–2.8) Men: 2.0 (1.4–3.1)	Age*¶, sex*, marital status*#, total number of partners (past year)*, condom use*, history of gonorrhoea, history of foreign partner, number of same sex partners, alcohol consumption, smoking*¶, history of illicit drug use#, occupational group¶
Sheringham, 2011 [23]	Chlamydia	OR: Screening tests among young women: 1.48 (1.37–1.60) Screening tests among young men: 2.13 (1.90–2.39)	AORs: Screening tests among young women: 1.41 (1.30–1.52) Screening tests among young men: 1.68 (1.49–1.89)	Age*, area-level deprivation (IMD)*, >1 sex partner (past year)*, screening setting
Simms, 2009 [24]	Chlamydia	OR: Screening tests among young women: 1.32 (1.22–1.42) Screening tests among young men: 2.02 (1.79–2.27)	AORs: Screening tests among young women: 1.37 (1.27–1.50) Screening tests among young men: 1.57 (1.37–1.80)	Age*, new sex partner (past 3 months)*, >1 sex partner (past year)*, screening setting*, phase of screening programme implementation
Wayal, 2017 [8]	Any STI diagnosis in the last 5 years	ORs: Women: 1.75 (0.73–4.20) Men: 3.22 (1.31–7.89)	AORs: Women: 1.71 (0.65–4.51) Men: 2.48 (1.05–5.88)	Age*, marital status^¶^, academic qualification, socio-economic status^#^, area-level deprivation (Index of Multiple Deprivation, IMD), binge drinking, recreational drug use in the last year^#^, depressive symptoms, number of sexual partners in the last 5 years*, <16 years at sexual debut^¶^, sexual competence at sexual debut^¶^, 10+ partners in the last 5 years*, concurrent partnerships in the last 5 years^¶^, paid for sex in last 5 years^#^, condomless sex with 2+ partners in the last year
***Association between ethnicity and coinfection/repeat infections***				
Dragovic, 2002 [12]	Gonorrhoea and Chlamydia	Not reported	Not reported—p-values only from the multivariable analysis	Age*, sex*, sexual orientation, country of birth
Hughes, 2001 [13]	Acute STIs	Not reported	HR: Men and women: 1.87 (1.63–2.13)	Gender and age-group*, history of previous STI*, sexual orientation*, number of recent sexual partners*, sex abroad in the past year, history of injecting drugs.
Hughes, 2013 [14]	Gonorrhoea	HR: Men and women: 1.77 (1.12–2.78)	HR: Men and women: 1.59 (0.96–2.63)	Year of first diagnosis*, age group among heterosexuals, age group among MSM#, sexual orientation*, resident in same town as clinic, area-level deprivation (IMD)*, reported history of gonorrhoea*, reported history of any STI, number of partners in last 3 months*, sex with a high risk sexual partner (past 12 months), currently a sex worker, non-completion of clinic’s behavioural pro-forma*, ever injected drugs

*Association with the STI examined;

^#^Associated with the STI examined only among men;

^¶^Associated with the STI examined only among women;

^†^Associated only with chlamydia in women and with gonorrhoea and chlamydia in men; Variables which are not marked with any of the symbols

*^#¶^ were either removed from multivariable logistic regression models because of lack of association in earlier iterations of the model, or were not statistically significant in the model which produced the adjusted odds ratios are presented in this table;

Abbreviations: IMD, Index of Multiple Deprivation; HR, Hazard Ratio(s); OR, Odds Ratio(s); IRR, Incidence Rate Ratio(s); CI, Confidence Interval; MSM, men who have sex with men

The greater risk of reinfection with acute STIs among BCs re-attending sexual health clinics compared to those of white/WB ethnicity was also not fully explained by differences in these two ethnic groups in the socio-demographic factors, behavioural risk factors, and history of previous STI diagnosis [13]. Another study conducted among sexual health clinic attendees showed that adjusting for sexual orientation, area-level deprivation, and history of gonorrhoea diagnosis explained the greater risk of repeat gonorrhoea infections observed among those of BC ethnicity compared to those of white/WB ethnicity [14]. With regards to the risk of co-infection with chlamydia and gonorrhoea, one study found that ethnic differences in age and sex (being <20 years and female) explained the higher risk of co-infection among BC people [12].

### Ethnic differences in the prevalence of behavioural factors associated with STIs

As shown in Table 2, regardless of study design and ethnicity, factors such as age, multiple partners, and condomless sex were associated with STI diagnosis among men and women [6–8, 21, 22]. Individual-level economic status [8], sexual orientation [6], recreational drug use [8, 22] and paying for sex [7, 8] were found to be associated with STI diagnosis only among men, whereas marital status [8, 22], smoking [22], having new sex partners from another country [7], concurrent partnerships, sexual debut <16 years, and lack of sexual competence at sexual debut [8] (sexual debut was considered as sexually competent if the study participants reported: an absence of duress and regret about timing; autonomy of decision; and that a reliable form of contraception was used) were associated with STI diagnosis only among women. Data on factors that influence STI risk behaviours were available only from one longitudinal school-based survey which showed that depressive symptoms, low family support, and substance use were the strongest predictors of reporting sexual debut <16 years, having more than one sex partner, or condomless sex [25].

The following section describes how behavioural factors associated with STIs varied for BCs relative to white/WB ethnic groups (Table 3).

**Table 3:** ^§^Ethnic variations in behavioural risk factors for STIs.

Behavioural risk factors	Authors	Men				Women			
		White (or white British) ethnicity	White (or white British) ethnicity	Black Caribbeans	Black Caribbeans	White (or white British) ethnicity	White (or white British) ethnicity	Black Caribbeans	Black Caribbeans
		N	%	N	%	N	%	N	%
**Sexual behaviours**									
***Age at sexual debut***									
<16	Fenton,2005 [7]*^#^	5596	27.9%	108	56.3%	5414	22.0%	116	22.3%
<16	Wayal, 2017 [8]*^#^	6096	26.7%	88	60.6%	6214	20.2%	102	21.2%
≤13	Jayakody, 2011 [25]	229	10%	78	35%	181	3%	78	5%
***Sexual competence at sexual debut***									
Yes (age-standardised)	Wayal, 2017 [8]*^#^	5745	47.4%	76	32.9%	5948	47.9%	97	40.9%
***Number of sex partners***									
In the last 5 years (5+ partners)	Fenton, 2005 [7]*^#^	5479	21.0%	106	35.7%	5311	11.9%	112	10.4%
In the last 5 years (5+ partners, age-standardised)	Wayal, 2017 [8]*^#^	5951	13.9%	83	27.1%	6103	8.3%	94	7.7%
2 or more partners ever (among 13-16-year-olds)	Jayakody, 2011 [25]	229	17%	78	57%	181	7%	78	11%
Ever had sex	Jayakody, 2011 [25]	229	31%	78	49%	181	16%	78	22%
***Concurrent partnerships***									
In the last year (among those reporting any partners in the past year)	Fenton,2005 [7]*^#^	4849	13.9%	90	25.4%	4774	8.8%	89	11.5%
In the last 5 years (age-standardised)	Wayal,2017 [8]*^#^	5349	14.8%	78	26.5%	5235	8.0%	76	9.1%
***Paid for sex***									
In the last 5 years (age-standardised)	Wayal, 2017 [8]*^#^	6002	3.1%	84	5.1%	6165	0.03%	96	0.0%
***Homosexual partnerships***									
In the past year (≥1 new homosexual partners)	Fenton, 2005 [7]*^#^	5568	1.4%	104	2.1%	5396	0.9%	115	1.0%
Ever had genital contact with same-sex partner (age-standardised)	Wayal, 2017 [8]*^#^	6130	5.7%	92	1.1%	6286	6.6%	102	2.2%
***Condom use/non-use***									
Condomless sex with >1 partner in the past year	Wayal, 2017 [8]*^#^	5906	7.4%	82	11.6%	6086	5.0%	96	5.9%
Ever had condomless sex	Jayakody, 2011 [25]	229	11%	78	12%	181	8%	78	8%
Condom use at last sex	Jayakody, 2011 [25]	61	76%	31	93%	28	68%	17	74%
***Partner(s) from outside the UK***									
New partner(s) from outside the UK (past 5 years)	Fenton, 2005 [7]*^#^	5072	13.2%	95	20.4%	5977	6.3%	99	18.1%
New partner(s) from outside the UK (past 5 years)	Wayal, 2017 [8]*^#^	5368	7.2%	79	5.8%	5255	3.5%	76	5.5%
**Substance use related factors**									
***Recreational drug use***									
In the last year (age-standardised)	Wayal, 2017 [8]*^#^	5934	15.6%	84	12.6%	6133	7.0%	91	11.2%
***Smoking***									
Current smoking (age-standardised)	Wayal, 2017 [8]*^#^	6151	26.5%	92	19.6%	6291	25.5%	105	21.4%

^§^N is the denominator for the ethnic group and % indicates the proportion of people from that ethnic groups that reported the behaviour;

^#^Distributions of numbers of sex partners are available in some papers. We present summary measures for brevity and to aid comparability between papers;

*Analyses were weighted by study authors, to account for unequal probability of selection for the survey.

Weighted denominators are presented.

#### Age and sexual competence at sexual debut

The national probability surveys show that compared to white/WB men, the proportion of BC men reporting early sexual debut, i.e., <16 years, was double (56.3%-60.6% vs. 26.7%-27.9%) [7, 8] and similarly, a London school-based survey estimated sexual debut ≤13 years to be 35.0% and 10.0% among BC and white/WB young men respectively [25]. The national survey also showed that the proportion of BC men reporting that they were sexually competent at sexual debut was lower (32.9%) than among white/WB men (47.4%) [8]. In contrast, among BC and white/WB women, the prevalence of reporting sexual debut <16 years was similar in the national probability surveys (~20.0%) [7, 8] as was the prevalence of sexual debut ≤13 years in the London-school based survey (~5.0%) [25]. The national survey showed that the reporting of sexual competence at sexual debut among BC women was lower (40.9%) than among WB women (47.9%) [8].

#### Partner numbers

In national probability surveys, the proportion of BC men reporting five or more partners in the last five years was higher (range: 27.1%-35.7%) than for white/WB men (range: 13.9%-21.0%) [7, 8], whereas the proportions were similar among BC and white/WB women (range: 7.7%-11.9%) [7, 8]. Similarly, among 13-16-year-olds in the London school-based survey, the proportion of BC men reporting two or more partners ever was higher (57.0%) than for white/WB young men (17%) but proportions were similar among BC and white/WB young women (11.0%, 7.0% respectively) [25]. Among these 13–16 year olds, the proportion of BC men (49.0%) and women (22.0%) reporting ‘ever’ having sex was higher than for white/WB men (31.0%) and women (16.0%).

#### Sexual behaviours

In national probability surveys, the proportion of BC men reporting concurrency in the last year among sexually active participants [7] or in the last five years [8] was higher (~26.0%) than for white/WB men (~14.0%); however, this difference was explained by differences in age between these two ethnic groups [8]. The proportions of BC and white/WB women reporting concurrency (8.0%-11.5%) did not vary by ethnicity. The proportions of BC and white/WB men reporting paying for sex in the last five years were and 5.1% and 3.1% respectively, and among women no BC women reported this behaviour and it was reported by 0.03% white/WB women [8]. There were minor differences in the proportions of BC and white/WB men reporting one or more new same-sex partnerships in the last year (2.1% and 1.4% respectively) and among women it was 1.0% and 0.9% respectively [7]. The proportions of BC men and women reporting ‘ever’ having genital contact with same-sex partners was lower (1.1% and 2.2% respectively) than for white/WB men and women (5.7% and 6.6% respectively) [8].

The national probability survey showed that the proportion of BC men reporting condomless sex with two or more partners in the last year was higher (11.6%) compared to white/WB men (7.4%) whereas the proportions of BC and WB women reporting this behaviour were similar (~ 6.0%) [8]. In the London school survey of 13–16 year olds [25], the proportions of men ‘ever’ having condomless sex were similar among BC and white/WB men (~12%) and women (8.0%). However, the proportion reporting condom use at last sex was higher among BC men and women (93.0% and 74.0%, respectively) than among white/WB men and women (76.0% and 68.0%, respectively) [25]. Likewise, the proportions reporting new sex partners from outside the UK in the past five years was higher among BC men and women (20.4% and 18.1%, respectively) than among white/WB men and women (13.2% and 6.3%, respectively) [7].

### Substance use

With regards to substance use, the national probability survey shows that similar proportions of BC and white/WB men and women reported recreational drug use in the past year (12.6% and 15.6% respectively in men and 11.2% and 7.0% respectively in women) [8]. Whereas BC men and women were less likely to report current smoking (19.6% and 21.4%) than white/WB men and women (26.5% and 25.5% respectively) [8].

### Ethnic differences in sexual healthcare seeking behaviour

Three studies (Table 4) reported data on variations in sexual healthcare seeking behaviours among BC and white/WB ethnic groups [7, 8, 26]. Both nationally representative surveys reported that a higher proportion of BC people attend sexual health clinics than white/WB people [7, 8]. Additionally, a clinic-based survey reported that symptomatic BC men were less likely to delay seeking care (i.e., waited more than seven days after symptoms started before seeking care) than white/WB men; however, the former were more likely to have multiple sex partners when symptomatic [26]. Among women, similar proportions of BC and white/WB women reported a delay in seeking care (44.4% and 48.1% respectively) or to have sex after symptoms started (49.2% and 54.2% respectively).

**Table 4:** ^§^Ethnic variations in sexual healthcare seeking behaviours.

Health seeking behaviours	Authors	Men				Women			
		White (or white British) ethnicity	White (or white British) ethnicity	Black Caribbeans	Black Caribbeans	White (or white British) ethnicity	White (or white British) ethnicity	Black Caribbeans	Black Caribbeans
		N	%	N	%	N	%	N	%
Ever attended sexual health clinic	Fenton,2005 [7]*	5341	14.1%	102	28.0%	5234	11.9%	109	38.5%
Ever attended sexual health clinic	Wayal, 2017 [8]*	5704	11.8%	80	23.6%	5922	12.7%	87	26.8%
Evidence that tried/used GP before attending clinic, among sexual health clinic attendees	Gerressu,2012 [26]	1093	23.5%	163	16.6%	1171	27.4%	181	26.0%
Delay in seeking care, among symptomatic sexual health clinic attenders (waited >7 days after symptoms started before seeking care)	Gerressu, 2012 [26]	275	45.1%	26	30.8%	310	48.1%	45	44.4%
Had sex since symptoms started, among symptomatic sexual health clinic attendees	Gerressu, 2012 [26]	333	38.1% with one partner; 9.3% with more than one partner	33	21.2% with one partner; 18.2% with more than one partner	406	54.2% with one partner; 6.6% with more than one partner	67	49.2% with one partner; 4.5% with more than one partner

*Analyses were weighted by study authors, to account for unequal probability of selection for the survey;

^§^N is the denominator for the ethnic group and % indicates the proportion of people from that ethnic groups that reported the behaviour.

Denominators in this table are weighted, but percentages were calculated using weighted data.

## Discussion

This is the first systematic review to examine factors influencing the sustained disproportionate burden of bacterial STIs and TV among people of BC ethnicity in the UK. Our findings highlight that in most studies, the higher risk of STI acquisition among BCs compared to white/WB ethnic group persisted after adjusting for various socio-demographic factors, behavioural risk factors, and substance use. Importantly, however, our review suggests that the higher prevalence of sexual risk behaviours, including early sexual debut, concurrency and larger partner numbers reported among BC men compared to white/WB men potentially contributes to their disproportionately high STIs rates. In contrast, as shown in Tables 2 and 3, the higher STI burden among BC women compared to white/WB women exists despite adjusting for known risk factors for STIs, and there are similarities in the reporting of the prevalence of risk behaviours among women from these two ethnic groups. These findings suggest that STI prevention efforts targeting behaviour change among BC men might be more effective in this population. Encouragingly, our review also suggests that people of BC ethnicity are more likely to access sexual health clinics than people of white/WB ethnicity, which has implications for delivering STI prevention interventions through sexual health clinics to reach this population. In terms of clinical practice, the limited existing evidence on co-infection and reinfection with bacterial STIs among BC people suggests that retesting following treatment and enhancing partner notification, especially among young women, could be beneficial for improving sexual health outcomes for this population group.

### Strengths & limitations

We included studies from the start of the electronic databases searched up to October 2016. The earliest study that met our inclusion criteria was published in 2000 [6], thereby reducing heterogeneity between studies in study populations over time. Sexual behaviour and its associated influences have changed little at a population-level since 2000 [27–28], but changes in the delivery of sexual healthcare in recent years are likely to have influenced sexual healthcare seeking [29]. The introduction of more sensitive STI testing techniques over time may also have influenced STI diagnosis rates, but their application is unlikely to have varied by ethnic group.

Our findings should be interpreted with caution given the variation in study methodologies which meant that we were unable to conduct a meta-analysis. For example, some studies used a generic ‘white ethnicity’ as the reference group while others specifically used WB ethnicity. Such conflation of people of white/WB ethnicity could have introduced bias especially in studies following the accession of ten central and eastern European countries to the European Union in 2004, resulting in increased numbers of people identifying as ‘white other’ [8, 30]. Differences in sexual behaviour between ‘white other’ and WB populations have also been reported [31]. The majority of the studies included in the review had limited internal and/or external validity, therefore the review results should be interpreted with caution, especially as most of the studies used data collected from sexual health clinic attendees in England. While we excluded studies that did not differentiate between people of BC ethnicity from other black ethnicities, we acknowledge that considerable heterogeneity exists within the BC ethnic group [32].

### Implications for future research and practice

Addressing inequalities is one of the priorities of the sexual health improvement framework in England [33]. Previous national and local policies have tended to prioritise HIV prevention to the exclusion of other STIs [34]. High rates of STI diagnoses in people of BC ethnicity have been highlighted since the early 2000 [11] yet there has been a relative dearth of studies addressing this issue. Our systematic review strengthens the evidence-base by enhancing understanding of the factors influencing ethnic differences in STIs which is vital for understanding research gaps and improving STI prevention interventions. It has been argued that STI prevention should focus on young people irrespective of ethnicity and account for gender differences [35–37] because patterning of risky and protective behaviours is mediated by ‘youth’ [25, 37]. However, this argument overlooks the role that ethnic identity may play in influencing STI risk.

The higher burden of STI diagnosis observed among BC women relative to white/WB women in the absence of behavioural differences highlight the need to conduct partnership-level studies of STI risk to inform STI prevention efforts. We did not find any qualitative studies conducted specifically among people of BC ethnicity. However qualitative studies of young people in the UK from major ethnic groups or of black ethnicity have shown that the broader social context, including religion [37], gender norms related to sex and sexuality influence partnership types and hamper condom use, exacerbating STI risk, especially among young women [34, 36, 37]. These studies have also highlighted a preference for same-ethnicity long-term partnerships but greater likelihood of disassortative mixing in casual partnerships [36, 37]. However, none of the studies included in our review examined the impact of sexual mixing patterns by ethnicity on STI risk. Better evidence is also needed on the impact of ethnicity related stigma and discrimination on STI related risk and healthcare seeking behaviours [35]. Mixed-methods research could improve our understanding of, for example, the role of partnership dynamics on STI risk among people of BC ethnicity and is currently underway [38].

## Supporting information

S1 Table Databases searched. (DOCX)

S2 Table Study screening questions. (DOCX)

S3 Table Quality appraisal of included studies. (DOCX)

S4 Table PRISMA checklist. (DOC)

S1 File Search strategy for systematic review (example: Search strategy for Medline). (DOCX)

S2 File Data extraction pro-forma for quantitative studies. (DOCX)
