# Supplementary material for: Understanding the burden of bacterial sexually transmitted infections and Trichomonas vaginalis among black Caribbeans in the United Kingdom: Findings from a systematic review
Source: PLoS One. 2018 Dec 7;13(12):e0208315. doi: 10.1371/journal.pone.0208315 (PMC6285827; doi:10.1371/journal.pone.0208315)
Supplement: S1 Table — (DOCX) [file pone.0208315.s001.docx]

# S1 Table. Databases searched

|  |  | ***Date searched*** |
| --- | --- | --- |
| 1 | Medline | 30/9/2016 |
| 2 | EMBASE | 2/10/2016 |
| 3 | CINHAL | 12/10/2016 |
| 4 | PsycINFO | 12/10/2016 |
| 5 | Scopus | 01/10/2016 |
| 6 | Web of sciences | 18/9/2016 |
| 7 | Via Proquest: British Humanities Index | 18/9/2016 |
| 8 | Via Proquest: Applied Social Science Index and Abstracts, | 18/9/2016 |
| 9 | Via Proquest: International Bibliography of the Social Sciences | 18/9/2016 |
| 10 | Via Proquest: Sociological Abstracts | 18/9/2016 |
| 11 | Cochrane:  - Database of systematic reviews  - CENTRAL  - DARE  - Cochrane methodology register  -HTA database | 2/10/2016 |
