# Supplementary material for: Understanding the burden of bacterial sexually transmitted infections and Trichomonas vaginalis among black Caribbeans in the United Kingdom: Findings from a systematic review
Source: PLoS One. 2018 Dec 7;13(12):e0208315. doi: 10.1371/journal.pone.0208315 (PMC6285827; doi:10.1371/journal.pone.0208315)
Supplement: S2 Table — (DOCX) [file pone.0208315.s002.docx]

# S2 Table. Study screening questions

*Note: continue to subsequent question only if the answer to the current question is “yes”*. *Otherwise exclude study from the review.*

|  | **Screening criteria** | **Decisions made** | **Total number of papers excluded due to *each* of these reason** |
| --- | --- | --- | --- |
| Qs 1 | Was the study published in English? | No: |  |
|  |  | Yes: |  |
| Qs 2 | Was the study conducted in the UK? | No: |  |
|  |  | Yes: |  |
| Qs 3 | Was the study about bacterial STIs and/or TV? | No: |  |
|  |  | Yes: |  |
| Qs 4 | Did the study examine any of the following among persons aged >14 years, who identified as black Caribbean compared to white/white British:   1. association between ethnicity (BC compared to white/WB) and bacterial STIs and/or TV controlling for known risk factors 2. ethnic variations in prevalence of risky sexual behaviours associated with STIs between these two ethnic groups 3. ethnic variations in healthcare seeking behaviours between these two ethnic groups and factors influencing it | No: |  |
|  |  | Yes: |  |
|  |  | Unclear: |  |
