# Supplementary material for: Understanding the burden of bacterial sexually transmitted infections and Trichomonas vaginalis among black Caribbeans in the United Kingdom: Findings from a systematic review
Source: PLoS One. 2018 Dec 7;13(12):e0208315. doi: 10.1371/journal.pone.0208315 (PMC6285827; doi:10.1371/journal.pone.0208315)
Supplement: S3 Table — (DOCX) [file pone.0208315.s003.docx]

# S3 Table. Quality appraisal of included studies


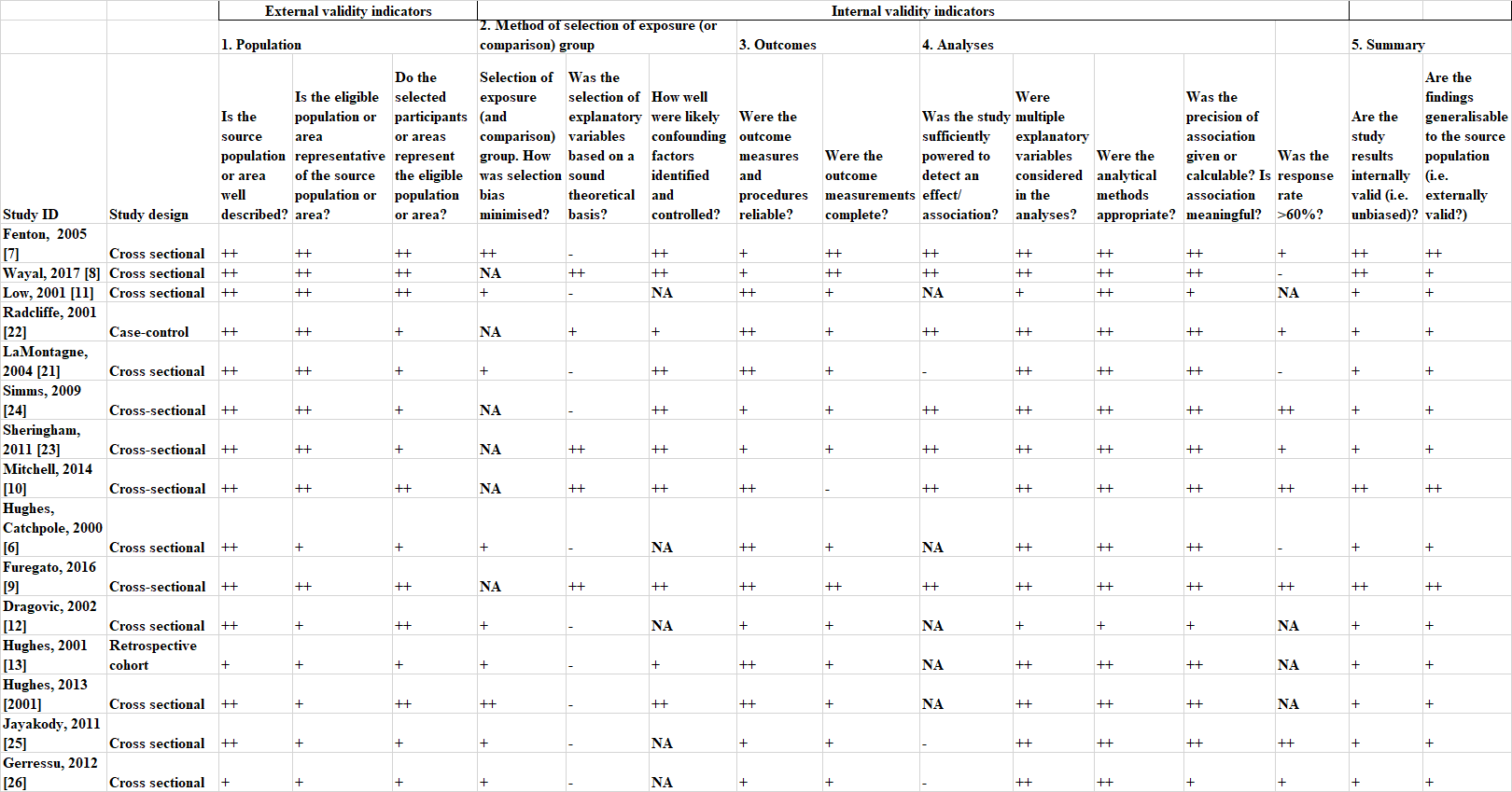
'++' indicates that for the stated external or internal validity checklist question, the study has been designed or conducted in such a way as to minimise the risk of bias; '+' indicates that either the answer to the checklist question is not clear from the way the study is reported, or that the study has not addressed all potential sources of bias for that particular aspect of study design; ' - ' indicates that the study design contains significant sources of bias; 'NR' stands for not reported and indicates that the study has not reported how they have (or might have) considered the aspects related to internal and/or external validity criterias; 'NA' stands for not applicable for the study design under review.
