# Supplementary material for: Understanding the burden of bacterial sexually transmitted infections and Trichomonas vaginalis among black Caribbeans in the United Kingdom: Findings from a systematic review
Source: PLoS One. 2018 Dec 7;13(12):e0208315. doi: 10.1371/journal.pone.0208315 (PMC6285827; doi:10.1371/journal.pone.0208315)
Supplement: S1 File — (DOCX) [file pone.0208315.s005.docx]

# S1 File. Search strategy for systematic review (example: search strategy for Medline)

1 African Continental Ancestry Group/ (34906)

2 exp Caribbean Region/eh [Ethnology] (3234)

3 ((black adj (caribbean or african)) or afro-caribbean or (black adj other)).mp. [mp=title, abstract, original title, name of substance word, subject heading word, keyword heading word, protocol supplementary concept word, rare disease supplementary concept word, unique identifier] (2502)

4 exp Ethnic Groups/eh, px, sn [Ethnology, Psychology, Statistics & Numerical Data] (41243)

5 ethnic*.mp. (123913)

6 1 or 2 or 3 or 4 or 5 (176584)

7 exp HIV Infections/ (246363)

8 (HIV* or human immunodeficiency virus or human immunedeficiency virus or human immuno-deficiency virus or human immune-deficiency virus or (human immun* adj deficiency virus) or acquired immunodeficiency syndrome or acquired immunedeficiency syndrome or acquired immuno-deficiency syndrome or acquired immune-deficiency syndrome or (acquired immun* adj deficiency syndrome)).mp. [mp=title, abstract, original title, name of substance word, subject heading word, keyword heading word, protocol supplementary concept word, rare disease supplementary concept word, unique identifier] (341833)

9 exp Sexually Transmitted Diseases/ (296239)

10 (sexually transmitted disease* or sexually transmissible disease* or sexually transmitted infection* or sexually transmissible infection* or sexually transmitted infectious disease* or sexually transmissible infectious disease* or sexually transmitted disorder* or sexually transmissible disorder* or STI or STD or genital ulcer* or genital infection* or genital disorder* or venereal or (sexually transmitted disease* adj3 (viral or bacterial or fungal or protozoan)) or herpes simplex or herpes genitalis or genital herpes or herpes virus or HSV* or gonorrhea or gonorrhoea or neisseria gonorrhoeae or gonococcal urethritis or gonococcal urethritis or gonococci or syphilis or treponema pallidum or chancre or primary syphilis or secondary syphilis or condylomata lata or candida albicans or monilia albicans or monilial infection or candidiasis or candida or candidal vaginitis or candidosis or vulvovaginitis or vulvitis or vulvovaginal candidiasis or vulvodynia or balanitis or lymphogranuloma venereum or chlamydia trachomatis or chlamydia infections or LGV or human papillomavirus or cervical cancer or HPV or genital wart* or anogenital wart* or anorectal wart* or anorectal wart* or penile wart* or condylomata acuminata or condyloma or bacterial vaginosis or gardnerella vaginalis or bacterial vaginitis or vaginitis or vaginosis).ti,ab. (231104)

11 exp Hepatitis, Viral, Human/ or Hepatitis/ (129155)

12 hepatitis*.ti,ab. (171224)

13 7 or 8 or 9 or 10 or 11 or 12 (744167)

14 exp Risk Factors/ (602242)

15 exp Behavioral Risk Factor Surveillance System/ (1290)

16 exp Risk-Taking/ (24868)

17 exp Risk Assessment/ (189415)

18 (risk or risky or incidence or prevalence).mp. [mp=title, abstract, original title, name of substance word, subject heading word, keyword heading word, protocol supplementary concept word, rare disease supplementary concept word, unique identifier] (2380353)

19 incidence/ or prevalence/ (371787)

20 epidemiology.mp. or Epidemiology/ (162659)

21 Qualitative Research/ (22427)

22 14 or 15 or 16 or 17 or 18 or 19 or 20 or 21 (2493506)

23 6 and 13 and 22 (8606)

24 exp Great Britain/ (311770)

25 ((great adj britain) or (united adj kingdom) or UK or england or ireland or wales or scotland).mp. [mp=title, abstract, original title, name of substance word, subject heading word, keyword heading word, protocol supplementary concept word, rare disease supplementary concept word, unique identifier] (454298)

26 24 or 25 (467394)

27 23 and 26 (482)
