# Supplementary material for: Understanding the burden of bacterial sexually transmitted infections and Trichomonas vaginalis among black Caribbeans in the United Kingdom: Findings from a systematic review
Source: PLoS One. 2018 Dec 7;13(12):e0208315. doi: 10.1371/journal.pone.0208315 (PMC6285827; doi:10.1371/journal.pone.0208315)
Supplement: S2 File — (DOCX) [file pone.0208315.s006.docx]

# S2 File. Data extraction pro-forma for quantitative studies

1. Authors and year, contact details
2. Aims
3. Study design

**Methods**

1. Setting
2. Study inclusion criteria
3. Exclusion criteria
4. Recruitment methods
5. Sampling method
   1. Convenience/consecutive/simple random/stratified random/multistage stratified random/other……………./unclear
   2. Refusal rate
   3. Loss to follow up (if applicable)
6. Type of STIs studied:
   1. Chlamydia
   2. Gonorrhoea
   3. Trichomonas
   4. Syphilis
7. Timing of outcome (last year etc)
8. Measurement type/diagnosis method: self-reported/biological testing
9. Participant characteristics
   1. Age: range, IQR, median, mean
   2. Gender
   3. Sexuality
   4. Ethnic group:
      1. White/ Black/Black African/Black Caribbean/Black Other/Mixed Black/Indian, Pakistani, Bangladeshi/Chinese/Other/Unclear
   5. HIV status
   6. Other: IDU/Pregnant
10. Description of target population
11. Description of source population
12. Total number of people included in the analysis
13. Response rate
14. How were missing data handled?
15. Statistical methods for analysis
16. Comparison of responders vs. non-responders, if reported;
17. Risk factor analysis and covariates included in the model of the analysis for each STI
    1. Univariate analysis:
       1. factors explored;
       2. factors significant, effect estimates (e.g. Odds ratios, 95% CI) and p-value of significance according to authors (p<0.05 or p<0.2) for each ethnic group compared
    2. Multivariate analysis:
       1. factors included;
       2. factors significant after adjusting significant, effect estimates (e.g. Odds ratios, 95% CI) and p-value of significance according to authors (p<0.05 or p<0.2) for each ethnic group compared
    3. Confounding variables
